# The Sumo proteome of proliferating and neuronal-differentiating cells reveals Utf1 among key Sumo targets involved in neurogenesis

**DOI:** 10.1038/s41419-021-03590-2

**Published:** 2021-03-22

**Authors:** Juan F. Correa-Vázquez, Francisco Juárez-Vicente, Pablo García-Gutiérrez, Sina V. Barysch, Frauke Melchior, Mario García-Domínguez

**Affiliations:** 1 Andalusian Centre for Molecular Biology and Regenerative Medicine-CABIMER, CSIC-Universidad de Sevilla-Universidad Pablo de Olavide, Av. Américo Vespucio 24, 41092 Seville, Spain; 2 Zentrum für Molekulare Biologie der Universität Heidelberg (ZMBH), Heidelberg University, DKFZ-ZMBH Alliance, Im Neuenheimer Feld 282, 69120 Heidelberg, Germany

**Keywords:** Enzyme mechanisms, Cell signalling, Chromatin, Sumoylation

## Abstract

Post-translational modification by covalent attachment of the Small ubiquitin-like modifier (Sumo) polypeptide regulates a multitude of processes in vertebrates. Despite demonstrated roles of Sumo in the development and function of the nervous system, the identification of key factors displaying a sumoylation-dependent activity during neurogenesis remains elusive. Through a SILAC (stable isotope labeling by/with amino acids in cell culture)-based proteomic approach, we have identified the Sumo proteome of the model cell line P19 under proliferation and neuronal differentiation conditions. More than 300 proteins were identified as putative Sumo targets differentially associated with one or the other condition. A group of proteins of interest were validated and investigated in functional studies. Among these, Utf1 was revealed as a new Sumo target. Gain-of-function experiments demonstrated marked differences between the effects on neurogenesis of overexpressing wild-type and sumoylation mutant versions of the selected proteins. While sumoylation of Prox1, Sall4a, Trim24, and Utf1 was associated with a positive effect on neurogenesis in P19 cells, sumoylation of Kctd15 was associated with a negative effect. Prox1, Sall4a, and Kctd15 were further analyzed in the vertebrate neural tube of living embryos, with similar results. Finally, a detailed analysis of Utf1 showed the sumoylation dependence of Utf1 function in controlling the expression of bivalent genes. Interestingly, this effect seems to rely on two mechanisms: sumoylation modulates binding of Utf1 to the chromatin and mediates recruitment of the messenger RNA-decapping enzyme Dcp1a through a conserved SIM (Sumo-interacting motif). Altogether, our results indicate that the combined sumoylation status of key proteins determines the proper progress of neurogenesis.

## Introduction

The Small ubiquitin-like modifier (Sumo) is a small polypeptide similar to ubiquitin, capable of covalently attaching to other proteins as a post-translational modifier^1^. Sumo regulates multiple processes in the eukaryotic cell, although it shows a prominent role in transcription repression^2^. Among the different Sumo species, Sumo1 normally appears conjugated to proteins, while a large proportion of Sumo2 and Sumo3 (designated as Sumo2/3 due to high homology and similar function) is free and rapidly conjugated to proteins in response to stress conditions^3^. Sumoylation occurs at lysine residues, often included in the consensus ΨKxE (Ψ, large hydrophobic residue). For conjugation, proteolytically mature Sumo is activated and transferred to the conjugating enzyme Ubc9, which finally attaches Sumo to target proteins, with the eventual concourse of a Sumo ligase. Sumo maturation and excision from targets are achieved by specific proteases.

Sumoylation is essential in vertebrates as evidenced by the *Ubc9* mutation at an early postimplantation stage in the mouse embryo^4^. Sumo government of relevant biological processes makes at present more and more evident the involvement of this modifier in a multitude of diseases^5^. In the nervous system, the association of Sumo with a variety of neurodegenerative diseases has been shown^6,7^. Besides this, sumoylation plays a fundamental role in the establishment of the synapse and has been revealed as a cytoprotective mechanism in response to severe stress-like ischemia^8,9^. The crucial role Sumo plays during development, including the development of the nervous system, is noticeable^10^. Developmental regulation of the Sumo machinery has been reported for the rodent brain^11,12^. However, the role of sumoylation of specific factors in the process of neuronal differentiation has not been deeply investigated. Neurogenesis involves the spatiotemporal deployment of a high number of transcription factors and other proteins subjected to strict regulation^13,14^. We have previously assigned roles in neurogenesis to the Sumo protease Senp7 and to sumoylated Braf35, a component of the LSD1-CoREST histone demethylase complex^15,16^, but a detailed landscape of key Sumo targets involved in primary events at early neurogenesis still lacks. To uncover relevant roles of Sumo at the onset of neuronal differentiation, we have determined and compared the Sumo proteomes of proliferating and neuronal-differentiating cells. We have identified a number of proteins whose effect on neurogenesis depends on Sumo attachment. Among these proteins, we have found the transcription factor Utf1, a new Sumo target whose transcription activity depends on sumoylation.

## Materials and methods

### Endogenous Sumo immunoprecipitation, SILAC analysis, and mass spectrometry

Endogenous Sumo immunoprecipitation (IP) was performed as described in great detail^17^. Hybridoma-producing monoclonal antibodies Sumo1 (clone 21C7) and Sumo2/3 (Clone 8A2), developed by Dr. Mike Matunis^18,19^, were obtained from the Developmental Studies Hybridoma Bank developed under the auspices of the NICHD and maintained by The University of Iowa, Department of Biology (Iowa City, IA). Cultivation and antibody purification was done as described in refs. ^17,20^. For the quantification of the Sumo proteome upon treatment/mass spectrometry, P19 cells were grown for 6–7 doublings (from 30 to 3600 cm^2^ culture dish surface, i.e., 24 15 wells in the end) in stable isotope labeling by/with amino acids in cell culture (SILAC) Dulbecco’s modified Eagle’s medium (DMEM) medium containing dialyzed fetal bovine serum (FBS) (dialyzed 3× against phosphate-buffered saline (PBS) through a 6–8000 MWCO (molecular weight cut-off) bag), 2 mM l-glutamine, and 146 µg/ml lysine and 86 µg/ml arginine (conditions under which no undesired amino acid conversion, such as proline to arginine, could be detected). One set of 24 plates contained “light” lysine and arginine and the other set of 24 plates contained D4-lysine and 13C-arginine. At 16–18 h before collecting the cells, they were serum-starved in SILAC DMEM medium containing only glutamine and the respective type of lysine and arginine. One set of cells was treated with all *trans* retinoic acid (RA) at 1 μM for 4 days and the other set was treated with vehicle. For large-scale Sumo-IPs, the cells were lysed in 350 µl 2× lysis buffer per plate and lysates from all 48 plates were combined. Sumo-IPs were performed as stated above and TCA precipitated eluates were loaded onto NuPAGE® Novex® Bis-Tris Mini Gels (4–20%) and stained with Coomassie Brilliant Blue. In-gel digestion was performed as described^21^ with minor modifications. Subsequently, peptide extraction and mass spectrometry were performed similarly to that in refs. ^17,20^. Two independent experiments were performed. In experiment 1, heavy labeling was used for proliferation conditions and light labeling for differentiation conditions, while in experiment 2 reverse labeling was used. We considered only proteins giving a SILAC ratio in both experiments, being the ratio in one experiment inverse to the ratio in the other experiment, both with a log_2_ value ≥|1|.

### Cell culture, transfection, flow cytometry, immunofluorescence, and embryo electroporation

Human 293T and mouse P19 cells were cultured in DMEM (Sigma-Aldrich, St. Louis, MO, USA) supplemented with 10% FBS (Sigma-Aldrich) and α-modified Eagle’s medium (Hyclone, Logan, UT, USA) supplemented with 7.5% calf (Hyclone) and 2.5% FBS, respectively, and 10 ml/l of an antibiotic solution with penicillin (100 U/ml) and streptomycin (10 mg/ml) (Sigma-Aldrich). P19 cells were directly obtained from ATCC (Teddington, Middlesex, UK). Cells were tested for the absence of mycoplasma. Transfections were performed with Lipofectamine 2000 (Invitrogen, Life Technologies, Paisley, UK) 36 h before harvesting the cells. All transfection constructs, except the GFP expression vector pEGFP_C2 (BD Biosciences Clontech, San Jose, CA, USA), were derived from vector pAdRSV-Sp^22^, in which different complementary DNAs (cDNAs) were cloned with Flag or HA tags. Mouse cDNAs were cloned by standard PCR procedures from a cDNA preparation from P19 cell-derived total RNA. PCR for cloning was performed with the Q5 polymerase (New England Biolabs, Ipswich, MA, USA). For GFP flow cytometry analysis, P19 cells were trypsinized after 24 h of transfection, resuspended in PBS, and analyzed in a BD FACSCalibur Flow Cytometer (BD Biosciences). Electroporation, preparation of embryos, and immunofluorescence on neural tube sections or P19 cells were performed as previously described^23,24^.

### Western blot, IP, pull-down, and yeast two-hybrid

Cell extracts were prepared in sodium dodecyl sulfate-containing denaturing buffer^17^, sonicated, immunoblotted with the Trans-Blot Turbo transfer system (Bio-Rad, Hercules, CA, USA), and analyzed using the Clarity Western ECL Substrate (Bio-Rad) in the Chemidoc XRS Imaging system (Bio-Rad). Brd2 antibody was as described in ref. ^25^. Commercial primary antibodies (1:1000) used were as follows: Atrx (sc-15408, Santa Cruz Btg., Dallas, TX, USA), Irf2bp1 (18847-1-AP, Proteintech, Manchester, UK), Morc3 (sc-83730, Santa Cruz Btg.), Prox1 (07-537, Upstate, Merck Millipore, Burlington, MA, USA), Sall4 (ab29112, Abcam, Cambridge, UK), Trim24 (14208-1-AP, Proteintech), Oct4 (H134, sc-9081, Santa Cruz Btg.), βIII-tubulin (ab18207, Abcam), α-tubulin (DM1A, T9026, Sigma-Aldrich), Kctd15 (PA5-25862, Thermo Fisher Scientific, Waltham, MA, USA), Utf1 (ab24273, Abcam), Dcp1a (WH0055802M6, Sigma-Aldrich), HA (H6908, Sigma-Aldrich), and Flag (M2, F1804, Sigma-Aldrich). Horseradish peroxidase-conjugated antibodies were from Sigma-Aldrich (1:10,000). Yeast two-hybrid assay was performed using the DUALhybrid Kit System (Biotech, Schlieren, Zurich, Switzerland), using the pLexA-N bait vector and the pGAD-HA prey vector. Production of proteins was performed in *Escherichia coli* DH5α strain and purification of GST fusion proteins was achieved by incubation with Gluthatione Sepharose 4B matrix (GE Healthcare, Buckinghamshire, UK). Sumo2 fusion was kept bound to the matrix, while Dcp1a was eluted by excision of the GST moiety through PreScission protease (GE Healthcare) incubation. Pull-down experiments were conducted as previously described^26^.

### RNA extraction, quantitative PCR, and chromatin IP

Total RNA was prepared with the RNeasy Mini Kit (Qiagen, Austin, TX, USA), using the RNase-Free DNase Set (Qiagen) on the column following the manufacturer’s instructions and cDNAs were synthesized using the iSCRIPT cDNA Synthesis Kit (Bio-Rad). Quantitative PCR (qPCR) was performed with Power SYBR Green on the 7500 Fast Real-Time PCR System (Applied Biosystems, Carlsbad, CA, USA). Chromatin IP (ChIP) was performed as previously described^27^. Relative quantities of gene expression levels were normalized to the expression of the housekeeping gene *Rplp0*. Primers are indicated in Supplementary Table S1.

### Statistical analysis

Two-tailed Student’s *t* test or one-way analysis of variance (*p* < 0.05) followed by Bonferroni post-test for multiple comparison were applied for statistical analysis of two groups (comparison with control) or more than two groups, respectively (*p < 0.05, **p < 0.01, ***p < 0.001). Normal distribution and similar variances were assumed. For expression analysis, three independent experiments were analyzed in triplicate. For cell counting, 150 cells from three independent experiments were analyzed. Represented values are means ± s.d. Results were double-blind assessed (group allocation and outcomes).

## Results

### Proteomic analysis identifies different subsets of Sumo targets associated with proliferation and neuronal differentiation

**Fig. 1:**
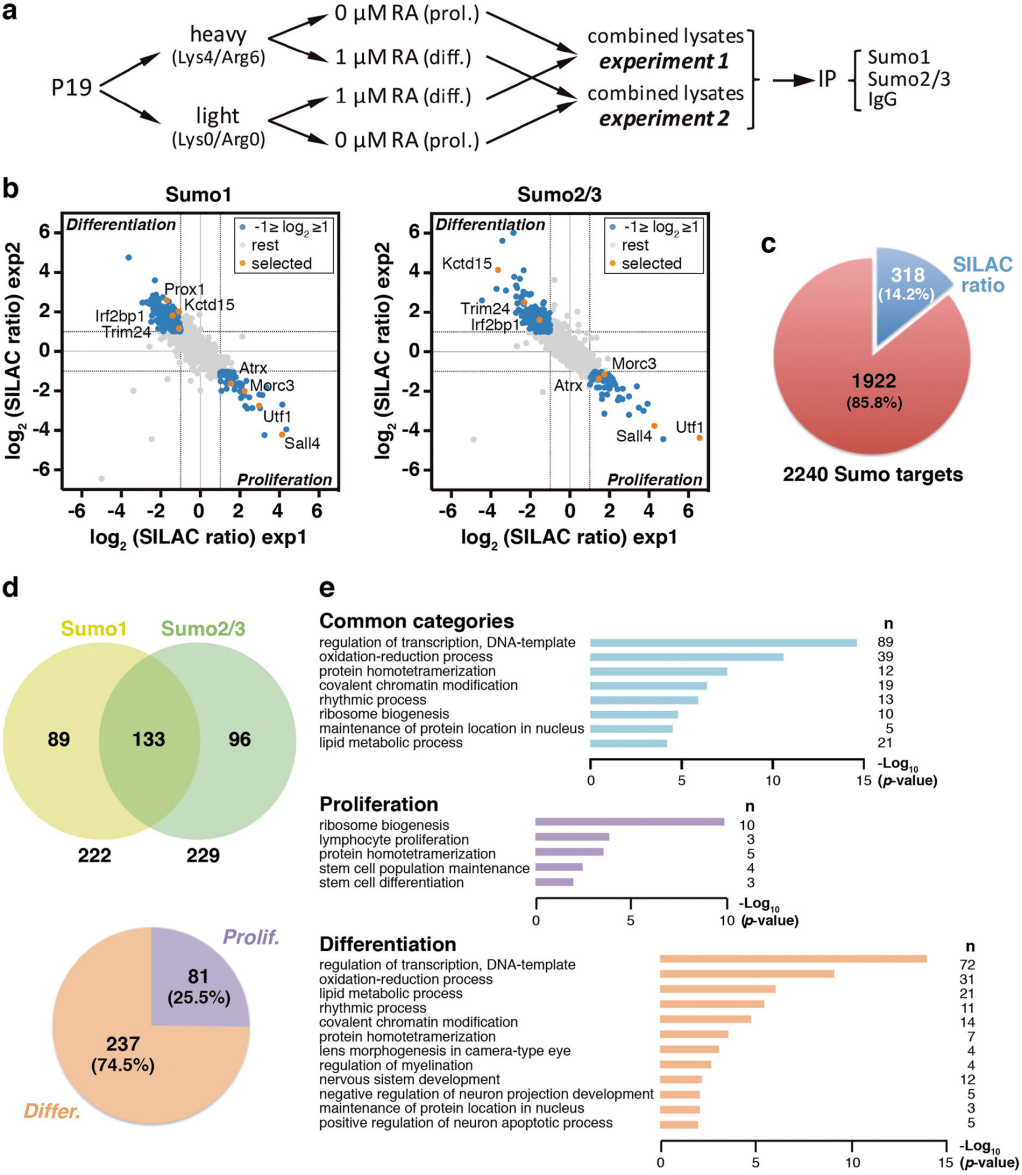
Induction of neuronal differentiation provokes changes in the Sumo proteome. **a** Schematic representation of the SILAC approach preceding MS analysis. Cells were labeled with heavy or light amino acids under proliferation (prol.) or retinoic acid (RA)-mediated differentiation (diff.), and lysates were combined for immunoprecipitation (IP). **b** Scatter plots of Sumo1 and Sumo2/3 putative targets showing a SILAC ratio in both experiments (experiment 1, exp1; experiment 2, exp2). Those with log_2_ (SILAC ratio) ≥1 in one experiment and a log_2_ (SILAC ratio) ≤−1 in the other experiment are in blue dots. Selected proteins for subsequent validation are in orange. The upper-left part of each plot includes proteins more sumoylated under differentiation conditions, while the lower-right part includes proteins more sumoylated under proliferation conditions. **c** Sector diagram showing the number of proteins indicated in blue in (**b**) in relation to the total number of Sumo targets identified. **d** Upper part, Venn diagram showing the number of proteins identified as Sumo1 or Sumo2/3 targets out of 318 proteins. Lower part, sector diagram showing the proportion of these proteins preferentially sumoylated under proliferation or differentiation conditions. **e** Gene ontology (GO) analysis of genes coding for the 318 proteins (common categories, *p* value <10^−4^), and GO analysis of genes coding for proliferation- or differentiation-associated targets (*p* value <10^−2^). *n* number of genes in each category.

To investigate the potential role of sumoylation during early neurogenesis, we aimed at identifying and comparing the Sumo proteomes of proliferating and neuronal-differentiating cells. P19 cells were used as a model due to easy manipulation and efficient differentiation, achieved through RA treatment or by forced expression of neurogenic factors^23,28^. For the identification of putative Sumo targets, we used the method described in refs. ^17,20^ to enrich endogenous sumoylated proteins, followed by SILAC-based quantitative mass spectrometry analysis. We compared cells under proliferation conditions (0 days of RA treatment) with cells treated with RA for 4 days, a stage in which they have formed embryoid bodies and start initial differentiation, mimicking early neurogenesis^29^. Combined cell lysates prepared under denaturing conditions to avoid de-sumoylation by endogenous Sumo proteases were subjected to IP with Sumo1 or Sumo2/3 antibodies or with normal mouse IgG. We performed two independent experiments. In experiment 1, we used heavy labeling for proliferation conditions and light labeling for differentiation conditions, while in experiment 2 we used reverse labeling (Fig. 1a). Mass spectrometry data (Dataset 1) permitted the identification of 2240 putative Sumo targets. To define candidates preferentially sumoylated under one or the other condition, we established the following criteria: we first considered only proteins giving an IP SILAC ratio in both experiments (1431 for Sumo1 and 1249 for Sumo2/3), then we asked that ratio in one experiment was inverse to the ratio in the other experiment, and finally, we considered only proteins with a log_2_ (ratio) ≥1 in one experiment and ≤−1 in the other experiment, that is, a fold change ≥2 in both experiments, with inverse ratios (Fig. 1b). Three hundred and eighteen proteins, with a variable preference for Sumo1 or Sumo2/3, fitted these criteria (Fig. 1c, d, upper panel). Note that this selection includes both proteins differentially sumoylated between conditions with no major changes in expression levels and proteins that are significantly better expressed in one or the other condition, in which they are sumoylated. Most of the proteins (74.5%) are preferentially associated with differentiation conditions (Fig. 1b, d, lower part). GO analysis indicated similar biological functions for putative Sumo1 and Sumo2/3 targets (Fig. 1e, common categories). Genes of proteins associated with proliferation was grouped into categories related to growth and pluripotency, while those associated with differentiation was grouped into categories related to transcription control, chromatin modification, oxidation–reduction metabolism, and nervous system development (Fig. 1e). We further classified the 318 candidates on the basis of their associated input SILAC ratios (Supplementary Table S2 and Supplementary Fig. S1a). One hundred and twenty-three proteins did not show input ratios in either experiment and were designated as group I. For the remaining 195 proteins, we calculated the relationship between the IP and input ratios. Those with a relation >1.5 or <0.67 in at least one experiment and at least for one Sumo paralog were designated as group II and considered that changes in sumoylation were greater than changes in expression. The rest of the proteins were designated as group III, and considered that changes in sumoylation were similar to changes in expression.

**Fig. 2:**
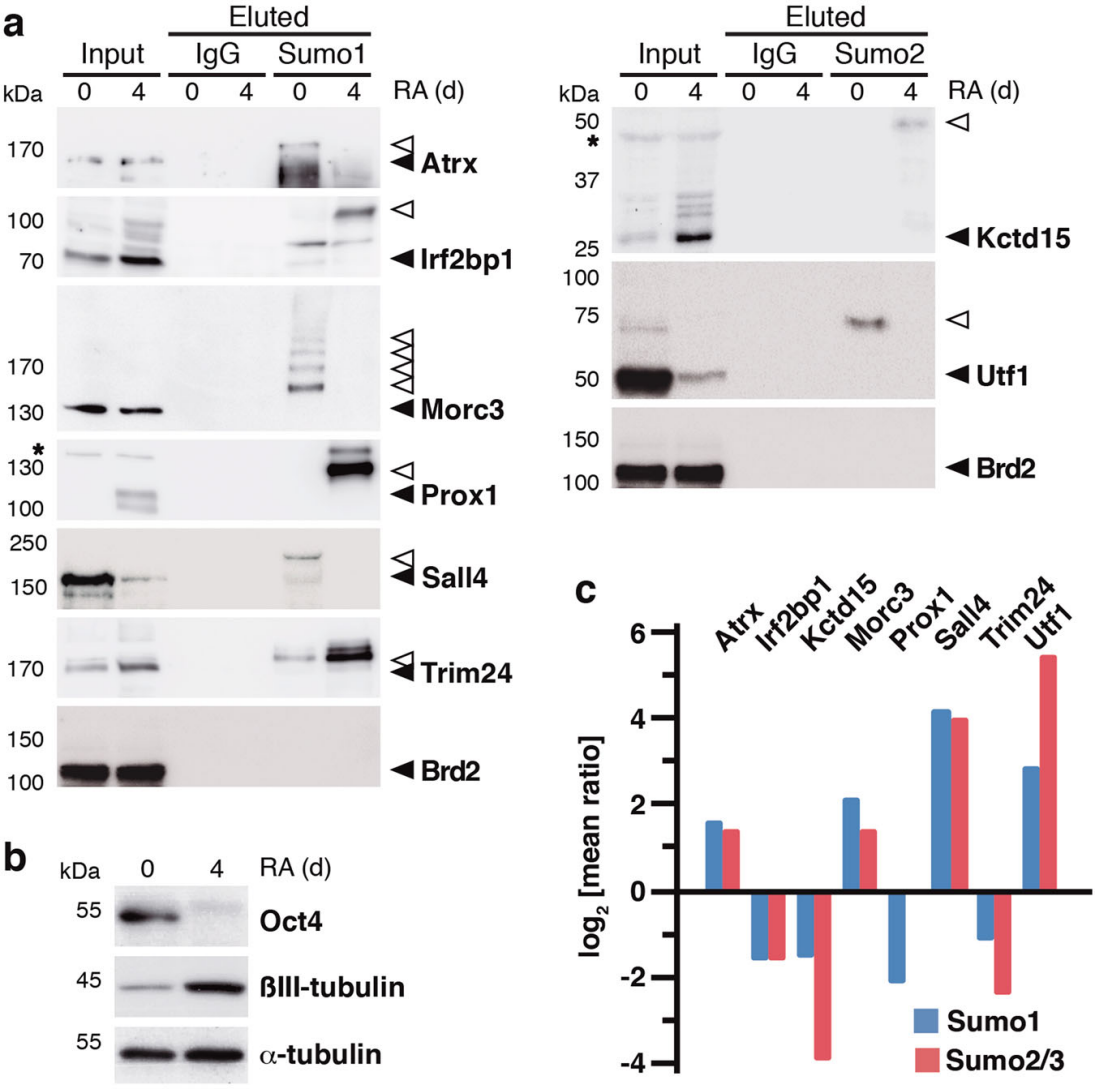
Selected Sumo targets differentially associated with proliferation and differentiation. **a** Validation of the proteomic results of a selection of proteins by western blot with specific antibodies against the different proteins, after precipitation with Sumo antibodies or with control IgG and peptide elution (100%), under proliferation (0 days (d)) or differentiation conditions (4 d of RA treatment). Some proteins were validated with Sumo1 antibodies and others with Sumo2/3 antibodies. Inputs (1.5%) are also shown. Black arrowheads indicate unmodified proteins, while white arrowheads indicate sumoylated products. Asterisks denote nonspecific bands. **b** Proliferation (0 days) and differentiation conditions (4 days of RA treatment) were checked by analysis of the pluripotency marker Oct4 and the differentiation marker βIII-tubulin. α-Tubulin was determined as a loading marker. **c** The log_2_ of the mean value of the ratio in experiment 1 and the inverse ratio in experiment 2 for selected proteins, either for Sumo1 (blue) or for Sumo2/3 (red), is represented.

To validate the proteomic approach, we analyzed the sumoylation of selected proteins (Fig. 1b, orange) by using specific antibodies against each protein. We selected proteins from group II and also some proteins from group I, based on the observed IP ratios and their putative functions. We confirmed the association of sumoylated Atrx, Morc3, Sall4, and Utf1 with proliferating cells, and of sumoylated Irf2bp1, Kctd15, Prox1, and Trim24 with differentiating cells (Fig. 2a, c). Analysis of Brd2 was included as a negative control. We monitored the differentiation process by checking the downregulation of the pluripotency marker Oct4 and upregulation of the neuronal marker βIII-tubulin (Fig. 2b).

### Expression of sumoylation mutants of selected proteins alters neuronal differentiation

Among validated proteins, we selected Kctd15, Prox1, Sall4, Trim24, and Utf1 for an initial characterization, based on previously described roles potentially involving them in governing the transition from proliferation to differentiation^30–34^. Of these proteins, Kctd15, Prox1, Sall4, and Trim24 were previously shown to be sumoylated^35–38^, while sumoylation of Utf1 has not been reported so far. Of note, Sall4 sumoylation has been previously shown for Sall4 isoform b^37^. However, in P19 cells we were only able to identify the longest isoform a. Sall4 size in western blot supports this (Fig. 2a). We confirmed in 293T cell lysates the sumoylation of expressed Flag- and HA-tagged versions of Sall4a and Utf1, respectively (Supplementary Fig. S2). Then, we proceeded to generate Lys to Arg (KR) sumoylation mutants of Utf1. Since software prediction indicated the absence of sumoylation consensus sites, we performed a detailed mutational analysis of the five Lys residues present in the mouse Utf1 sequence, which revealed the need to mutate Lys50, 119, and 210, to efficiently prevent sumoylation (Supplementary Fig. S2). Sall4a mutations in Lys151, 379, and 846 were according to ref. ^37^, and it completely abrogated sumoylation (Supplementary Fig. S2). We also generated expression constructs of wild-type (WT) and KR versions of the other proteins, with mutations according to refs. ^35,36,38,39^. Based on flow cytometry analysis of cells co-transfected with a GFP expression vector and the different expression constructs, transfection efficiency in P19 cells was 74.15 ± 2.8% (mean ± s.d., *n* = 10). We checked for similar levels of expressed tagged WT and KR versions of the proteins, for unaltered cell localization, and for overexpression levels in relation to the endogenous protein (Supplementary Fig. S3).

**Fig. 3:**
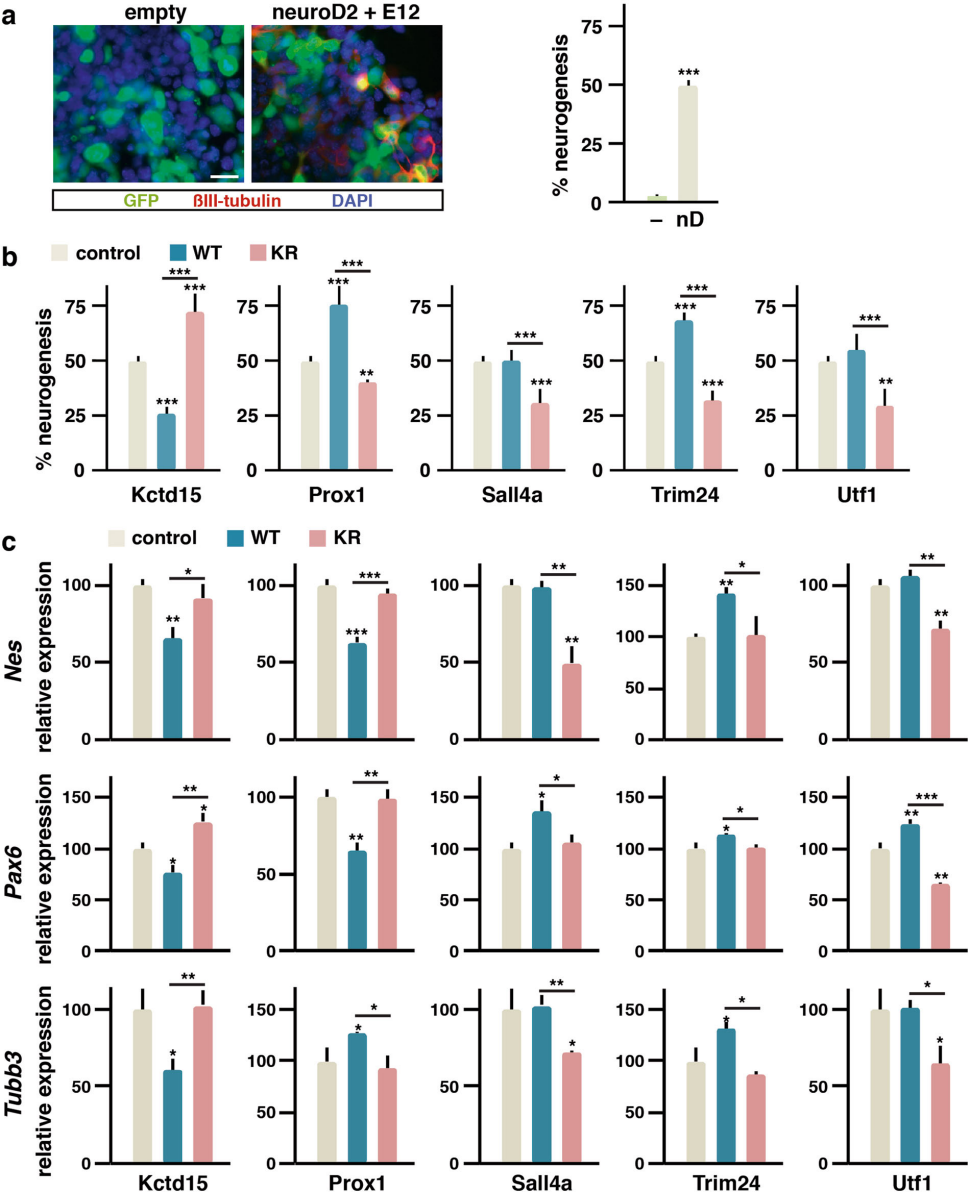
Expression of sumoylation mutants of key Sumo targets alters neurogenesis. **a** About 50% of neurogenesis is achieved in P19 cells after forced expression of the neurogenic factor NeuroD2 and the E12 co-factor (nD) for 72 h, in comparison with empty vector transfection (–), as determined in immunofluorescence experiments by the percentage of cells expressing the neuronal marker βIII-tubulin (red) among transfected cells (green, GFP positive). Nuclei were visualized by DAPI staining (blue). Scale bar 25 µm. **b** Quantification of the percentage of induced neurogenesis in the presence of expressed wild-type (WT) or sumoylation mutant (KR) versions of the indicated proteins. Control corresponds to the percentage of induced neurogenesis in (**a**). Values are means ± s.d. from counting 150 cells in six separated fields from three independent experiments. **c** Expression of early (*Nes* and *Pax6*) and late (*Tubb3*) neurogenesis markers was assessed in P19 cells by quantitative PCR after 48 h of RA treatment in the absence (control, empty vector) or the presence of expressed WT or KR mutant versions of the indicated proteins. Values are means ± s.d. from three independent experiments analyzed in triplicate. Statistical significance in relation to the control is indicated on top of each bar and other comparisons are indicated with a line. Statistical significance was determined by the Student’s *t* test. *p < 0.05, **p < 0.01, and ***p < 0.001.

We decided to study the effects on neurogenesis of KR versus WT versions of selected proteins in gain-of-function experiments. This approach, although involving ectopic expression, helps us to have an idea about the impact sumoylation can have on the activity of Sumo targets. We estimated neurogenesis in P19 cells according to previous reports^23,27^. Thus, we transfected different constructs together with expression vectors for the neurogenic factor NeuroD2 and its co-factor E12. Around 50% of cells expressed the neuronal marker βIII-tubulin following expression of neurogenic factor, while virtually none of the cells expressed this marker in the absence of neurogenic factor (Fig. 3a). The effect of the different constructs was variable, although the effects of WT and KR versions notably differed in all the cases (Fig. 3b and Supplementary Fig. S4). In general, KR versions displayed a negative effect, excepting Kctd15-KR, which enhanced neurogenesis. On the other hand, WT Prox1 and Trim24 enhanced neurogenesis, while WT Kctd15 impaired it. WT Sall4a and Utf1 did not affect NeuroD2-promoted differentiation.

We also tested the effect of the different constructs on transcription of differentiation markers by qPCR (Fig. 3c). We analyzed the expression of early markers *Nes* and *Pax6* and of later marker *Tubb3*. For convenience, we chose to treat the cells with RA for 48 h to promote differentiation. Results were according to those shown in Fig. 3b, with few exceptions: in line with the above results, WT Prox1 promoted *Tubb3* upregulation, but *Nes* and *Pax6* were downregulated, probably due to Prox1-mediated stimulation of neurogenesis negatively affecting early markers at the time of analysis.

### Effect of ectopic Kctd15, Prox1, and Sall4a in the developing neural tube depends on sumoylation

**Fig. 4:**
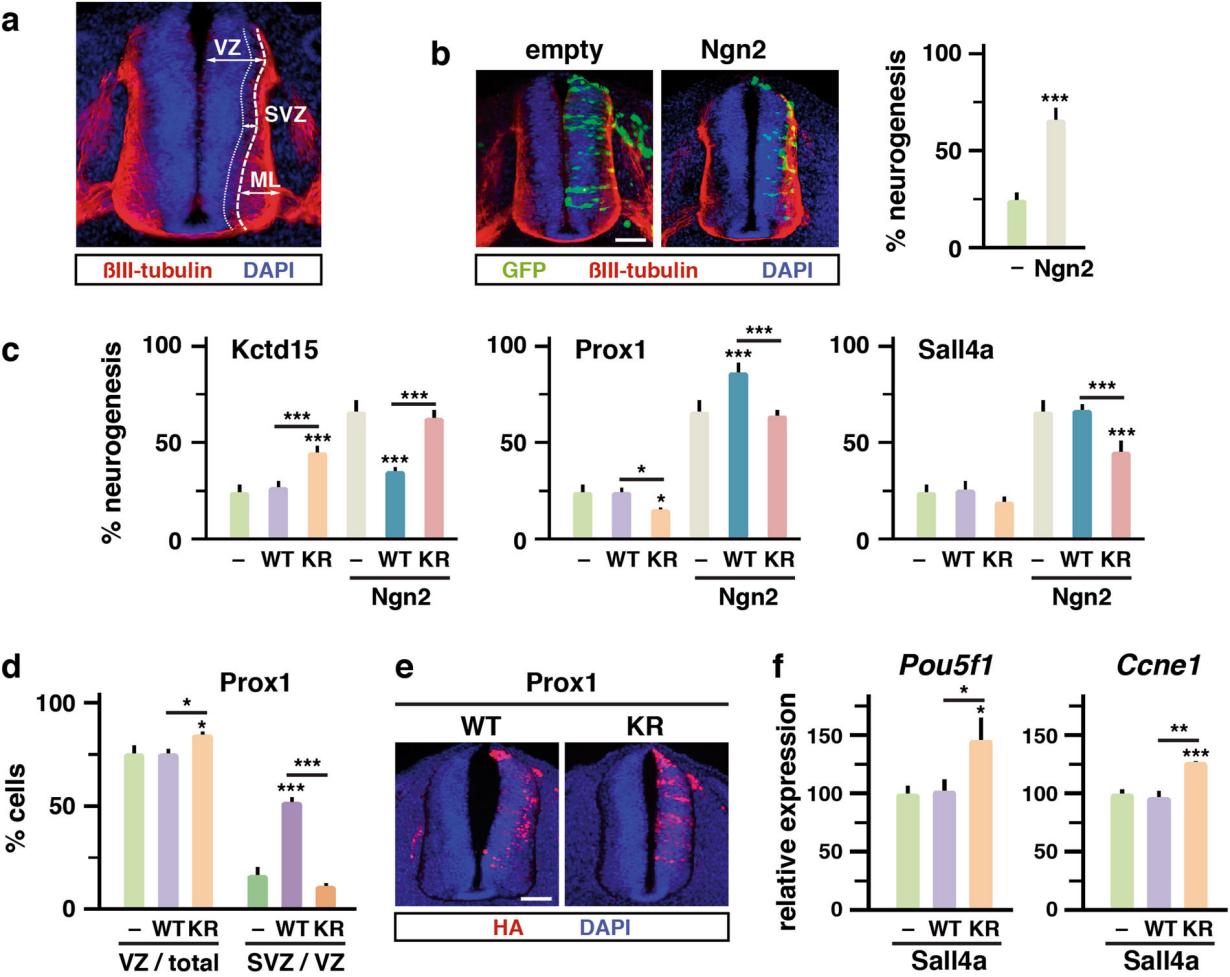
Kctd15, Sall4a, and Prox1 display altered effects in the neural tube when mutated for sumoylation. **a** Section of the developing neural tube indicating the limit between the proliferative ventricular zone (VZ) and the differentiation mantle layer (ML). The subventricular zone (SVZ) of migrating early postmitotic neurons at the pial surface of the VZ is also indicated. The ML-associated neuronal marker βIII-tubulin (red) and DAPI-stained nuclei (blue) are shown. **b** About 65% of neurogenesis is achieved in the developing neural tube after forced expression of the neurogenic factor Neurogenin 2 (Ngn2) for 30 h, in comparison with 25% of naturally occurring neurogenesis when the empty vector is electroporated (–). Neurogenesis was determined in immunofluorescence experiments by the percentage of transfected cells (GFP positive, green) located in the ML. **c** Effect on neurogenesis of WT or KR versions of the indicated proteins, in the absence (empty) or the presence of Ngn2. Controls (–) correspond to the percentages of neurogenesis in the absence (empty) or the presence of Ngn2 as determined in (**b**). **d** The percentages of control cells (empty vector electroporation), and of cells expressing WT or KR Prox1 in the absence of Ngn2, localizing to the VZ in relation to the total number of transfected cells, and localizing to the SVZ in relation to the cells localizing to the VZ, were represented. **e** Localization of expressed HA-tagged WT or KR Prox1 was analyzed by immunofluorescence with anti-HA antibodies (red). Nuclei were visualized by DAPI staining (blue). Scale bars 50 µm. **f** Expression of *Pou5f1* and *Ccne1* was assessed in P19 cells by quantitative PCR under proliferation conditions in the absence (–, empty vector) or the presence of expressed WT or KR versions of Sall4a. Represented values are means ± s.d. from counting 150 cells in five sections from three independent experiments (**b**–**d**) or from three independent experiments analyzed in triplicate (**f**). Statistical significance in relation to the control is indicated on top of each bar, other comparisons are indicated with a line. Statistical significance was determined by ANOVA (*p* < 0.0001), followed by Bonferroni’s post-test (95% confidence intervals) (**c**, **d**) or by the Student’s *t* test. *p < 0.05, **p < 0.01**, and ***p < 0.001 (**b**, **f**).

Since Kctd15, Prox1, and Sall4-like proteins have been previously involved in aspects of nervous system development in the vertebrate embryo^30,31,40^, we decided to analyze how sumoylation of these proteins affects the activity they display when expressed in the neural tube. During development, neural progenitors in the neural tube proliferate close to the lumen, the ventricular zone (VZ). They progressively exit the cell cycle and migrate into the pial surface or mantle layer (ML), a βIII-tubulin-positive region where they install to complete differentiation. Migrating early postmitotic neurons delimit a middle zone between VZ and ML, the subventricular zone (SVZ) (Fig. 4a). We turned to the technique of electroporation of the chick embryo, enabling transfection of expression constructs in neural progenitors of one-half of the neural tube. Around 25% of electroporated progenitors in the VZ naturally exit the cell cycle and migrate to the ML (Fig. 4b). However, following ectopic expression of the neurogenic factor Neurogenin 2 (Ngn2), 65% of electroporated cells localized to the ML (Fig. 4b). As in P19 cells, significant differences were observed between the effects of WT and KR versions when co-expressed with Ngn2 (Fig. 4c and Supplementary Fig. S5), and similarly, sumoylation of Prox1 and Sall4a associated with neurogenesis stimulation, while sumoylation of Kctd15 associated with neurogenesis impairment. Interestingly, Kctd15-KR promoted neurogenesis in the absence of ectopic Ngn2 (Fig. 4c).

Prox1 has been described to be expressed in the SVZ and to drive neural progenitors out of the cell cycle when ectopically expressed, but not to induce differentiation^31^. In the absence of Ngn2, Prox1-KR provoked a slight decrease of cells in the ML (Fig. 4c) and thereby a slight increase in the VZ (Fig. 4d), while Prox1-WT seemed not to have effects in comparison with control conditions. However, when looking at cell distribution in the VZ, we observed a clear effect of Prox1-WT in driving cells to the SVZ, and remarkably, this effect was abolished when impeding sumoylation (Fig. 4d). We confirmed the differential distribution of WT and KR versions of Prox1 by revealing the presence of the expressed constructs (Fig. 4e).

In *Xenopus*, it has been described that Sall4 promotes posterior neural fate by repression of pluripotency *pou5f3* family genes, the closest homologs of mammalian *Pou5f1* (Oct4)^40^. Thus, we wondered whether Sall4a might have a similar effect in our model and whether this effect depends on sumoylation. For that, we monitored *Pou5f1* expression in proliferating P19 cells (not forced to differentiate) in control conditions and in the presence of WT and KR versions of Sall4a. Interestingly, expression of the sumoylation mutant led to the increased expression of *Pouf51* and *Ccne1* (cyclin E1) (Fig. 4f), which may explain impaired differentiation by this mutant.

### Sumoylation controls transcriptional activity of Utf1

**Fig. 5:**
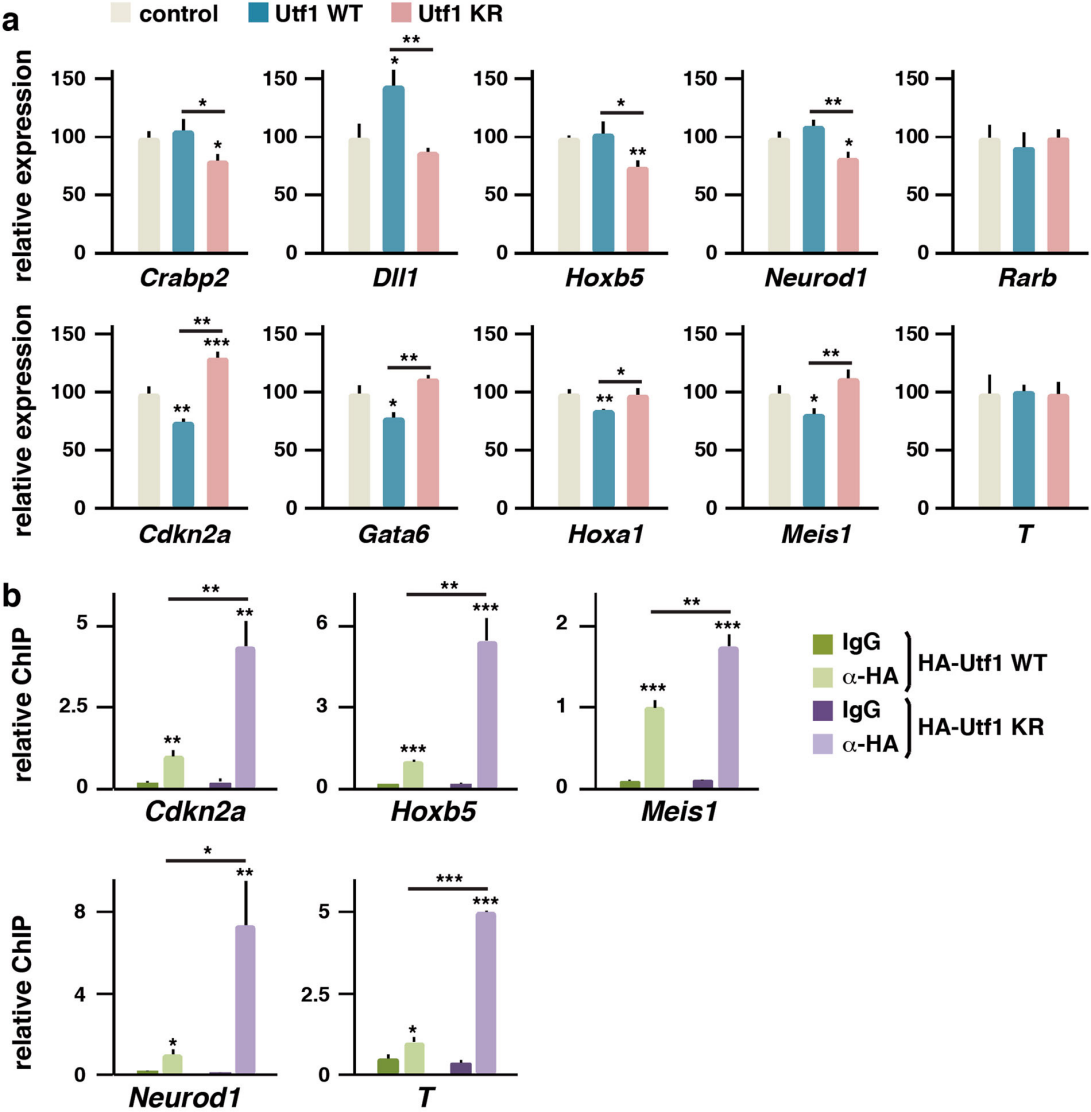
Sumoylation of Utf1 modulates chromatin affinity. **a** Expression of the indicated bivalent genes was assessed in P19 cells by quantitative PCR after 48 h of RA treatment in the absence (control, empty vector) or the presence of expressed WT or KR mutant versions of Utf1. **b** Localization of expressed HA-tagged WT or KR Utf1 to the indicated promoters was assessed by chromatin immunoprecipitation. IgG controls were established for each expressed construction. Statistical significance in relation to the control (**a**) or in relation to the corresponding IgG determination (**b**) is indicated on top of each bar, other comparisons are indicated with a line. Statistical significance was determined by the Student’s *t* test. *p < 0.05, **p < 0.01, and ***p < 0.001.

Utf1 has been described to control pluripotency and differentiation of embryonic stem (ES) and embryonal carcinoma (EC) cells^41,42^. As Utf1 seems restricted to eutherian mammals and it is absent from other vertebrates^43^, we turned to our murine EC model (P19) for detailed analysis. A dual role has been ascribed to Utf1 in the maintenance of the poised state of bivalent genes: on the one hand, its localization to the chromatin limits access of repressor complexes to promoters; on the other hand, Utf1 binds to the messenger RNA (mRNA)-Decapping enzyme Dcp1a, recruiting it to promoters, for degradation of leakage mRNAs^44^. Thus, we wondered about the impact of Utf1 sumoylation in the control of bivalent gene expression. We first identified a number of bivalent genes regulated by RA treatment in P19 cells (Supplementary Fig. S6). We then analyzed the effect of overexpressing WT and KR versions of Utf1 on the expression levels of bivalent genes in RA-treated P19 cells. As shown in Fig. 5a, in all the cases, marked differences were observed between the effects of WT and KR proteins, except on *T* (Brachyury), which was downregulated by RA (Supplementary Fig. S6), and on *Rarb*, which is not bound by Utf1^44^ and was used as a control. Intriguingly, for a set of genes, the WT protein was associated with a positive effect on gene expression in relation to the KR version, while for the other set of genes, it was the opposite (Fig. 5a). We next tested some genes from these two different sets, together with *T*, for localization of WT and KR versions of Utf1 to the corresponding promoters. For this, we conducted ChIP by precipitating the expressed HA-tagged proteins. Interestingly, we observed that, regardless of the effect on transcription, the sumoylation-deficient mutant bound more strongly to all promoters tested (Fig. 5b). This suggests that sumoylation modulates chromatin association of Utf1.

**Fig. 6:**
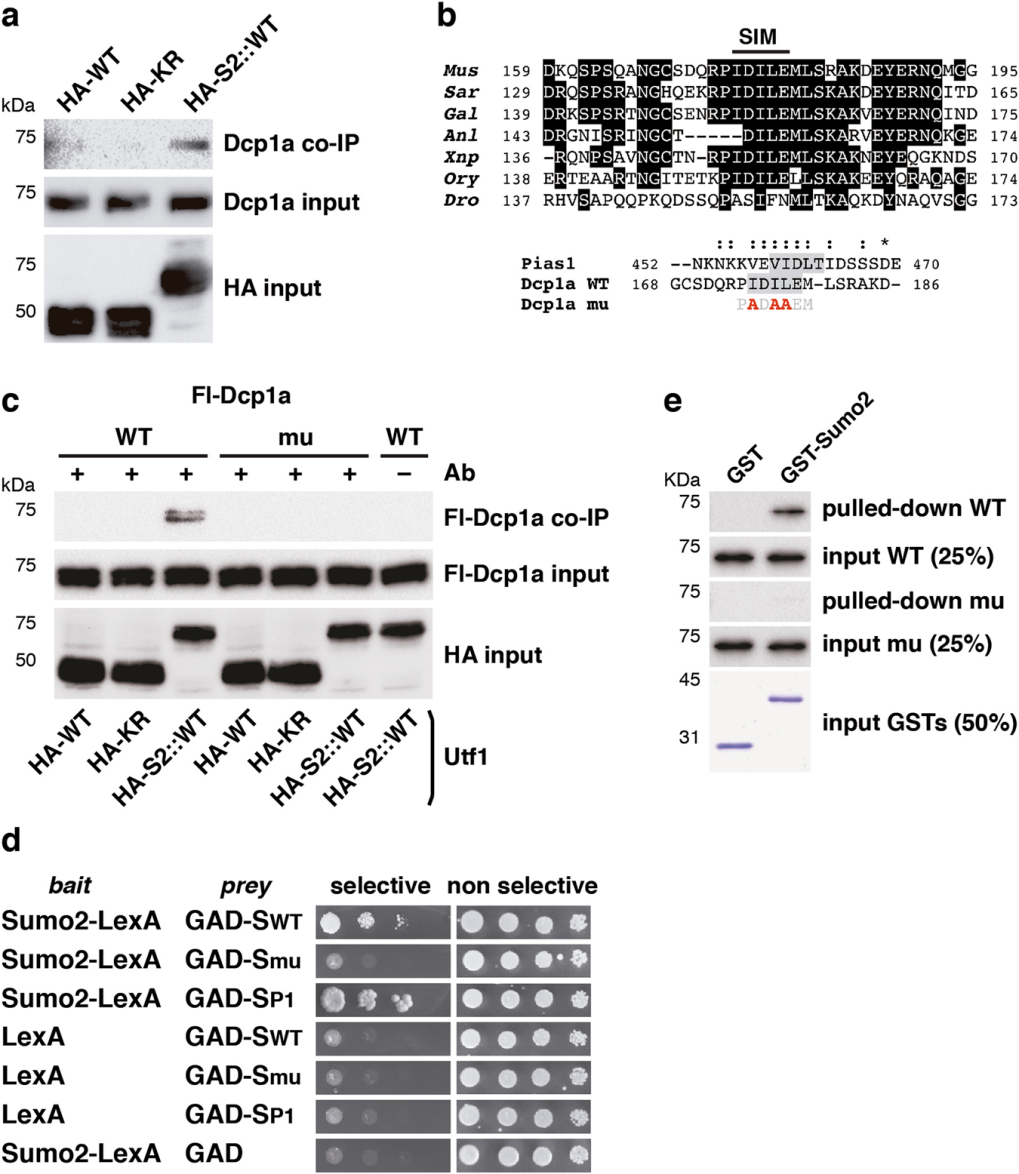
A conserved SIM in Dcp1a mediates interaction with sumoylated Utf1. **a** Interaction of endogenous Dcp1a with expressed HA-tagged WT, KR, or an N-terminal Sumo2 fusion of WT Utf1 (HA-S2::WT) was investigated by co-immunoprecipitation with anti-HA antibodies. Inputs (10%) are also shown. **b** Alignment of Dcp1a sequences from different species corresponding to a region with a predicted SIM (top, residues conserved in four out of the seven sequences have been boxed in black), and alignment of the mouse Dcp1a WT SIM with the mouse Pias1 SIM (gray shadow), indicating mutations (red) introduced in Dcp1a to disrupt the SIM (Dcp1a mu) (bottom). Similar and identical residues are marked with : and *, respectively. Protein accession numbers: *Mus*, *Mus musculus* (NP_598522.3); *Sar*, *Sarcophilus harrisii* (XP_012399256.2); *Gal*, *Gallus gallus* (XP_004944623.1); *Anl*, *Anolis carolinensis* (XP_008103449.1); *Xnp*, *Xenopus laevis* (XP_018096411.1); *Ory*, *Oryzias latipes* (XP_011475154.1); *Dro*, *Drosophila melanogaster* (NP_611842.1); Pias1, *Mus musculus* (XP_006511365.1). **c** Interaction of Flag (Fl)-tagged Dcp1a, wild type, or mutated in the SIM, with HA-tagged WT, KR, or an N-terminal Sumo2 fusion of WT Utf1 (HA-S2::WT) was investigated by co-immunoprecipitation with anti-HA antibodies. Inputs (10%) are also shown. **d** Two-hybrid assay to probe interaction between Sumo2 fused to the LexA DNA-binding domain (LexA) bait protein and WT (SWT) or mutant (Smu) version of the Dcp1a SIM or the Pias1 SIM (SP1) fused to the GAL4 activation domain (GAD) prey proteins. Interaction, as determined by yeast growth, was assessed both in selective and non-selective media. **e** Flag-tagged wild-type (WT) or SIM mutant Dcp1a-purified proteins were incubated with GST or a GST-Sumo2 fusion protein. Pulled-down products and the corresponding input were revealed by western blot. GST proteins were revealed by Coomassie Blue staining.

Finally, we decided to analyze the involvement of Utf1 sumoylation in Dcp1a recruitment. To this purpose, we conducted IP experiments by precipitating different HA-tagged expressed versions of Utf1 to analyze co-precipitated endogenous Dcp1a. In addition to WT and KR construct, we prepared a fusion construct of Utf1 with an N-terminal Sumo2 moiety, not cleavable by Sumo proteases. IP demonstrated preferred precipitation of Dcp1a with the Sumo2 fusion (Fig. 6a). Noticeably, analysis of the mouse Dcp1a sequence with the GPS-SUMO 1.0 tool^45^ revealed the presence of a putative Sumo-interacting motif (SIM). This SIM appeared well conserved among vertebrate Dcp1a proteins and displayed scores quite similar to classical Pias1 SIM^46^ (Fig. 6b). We then tested the interaction of the different HA-tagged Utf1 constructs with expressed Flag-tagged Dcp1a, WT or mutated in the SIM (relevant large hydrophobic residues replaced by alanine). Co-immunoprecipitation experiments revealed interaction of the Sumo2 fusion with WT, but not with mutated Dcp1a (Fig. 6c). We further tested the interaction of the Dcp1a SIM with Sumo2 by a two-hybrid approach. We prepared a bait construct of Sumo2 and prey constructs of the Dcp1a SIM in WT and mutant versions. Growth of yeast in selective medium revealed Sumo2 interaction with WT SIM but not with the mutant version (Fig. 6d). As a positive control, we tested the Pias1 SIM. We finally confirmed this interaction through a pull-down approach (Fig. 6e). Together, these findings indicate that Utf1–Dcp1a interaction can be regulated by sumoylation.

## Discussion

To get insights into the role that sumoylation plays during early neurogenesis, we have identified and compared the Sumo proteome of proliferating and differentiating cells. Our analysis indicates that a specific pattern of sumoylated proteins differentially defines each of these two conditions. Most identified putative Sumo targets consisted in transcription factors and chromatin-associated proteins, which confirms the prevalent transcriptional role of Sumo in regulating relevant cellular processes^47^. Interestingly, a higher number of sumoylated proteins (ca. 3-fold) associated with differentiation in relation to proliferation, suggesting that Sumo-mediated regulation of a high number of factors is required to initiate such a complex process. One hundred and twenty-three of the 318 proteins identified as differentially sumoylated, did not present input values in the proteomic analysis (group I). Among the rest, we distinguished between those with changes in sumoylation greater than changes in expression (group II) and those with similar changes in sumoylation and expression (group III). Interestingly, GO analysis indicates that genes coding for group II proteins are related to transcription, chromatin, and ribosome biogenesis, while those coding for group III proteins are related to different metabolic processes (Supplementary Fig. S1b). Similar observations have been indicated in a different proteomic study^48^.

In gain-of-function experiments, we have systematically observed significant differences between WT and KR versions of all proteins on all analyzed markers in the context of neurogenesis. The absence of dramatic effects in some cases may probably be due in part to technical limitations, but also might indicate that sumoylation is involved in fine-tuning of protein activities. Thus, the observation of major effects will require global affection of sumoylation or combined sumoylation deficiency of a number of related factors. Indeed, we have previously shown how globally altered sumoylation by overexpression of Sumo1 or Sumo2 in the developing neural tube has drastic consequences on neurogenesis^16^. Results from the forced expression of sumoylation mutants have been essential in raising our conclusions. In some cases, these mutants lacked the effect displayed by the corresponding WT version, indicating that effects depend on sumoylation. However, for certain proteins, the KR version behaved as a dominant negative, provoking a significant effect in comparison with control conditions, which probably indicates a competition with the endogenous sumoylable protein.

In general, we have been able to associate sumoylation of selected proteins with the progression of neurogenesis. Some of the best Sumo targets that we have identified here are proteins that are upregulated under differentiation conditions and which contribute to neurogenesis. Our findings that they are strong Sumo targets indicate that regulation by Sumo could be an important concept in the temporal progression of neurogenesis. Prox1 was more expressed under differentiation conditions, correlating its sumoylation with a positive role in neurogenesis. According to a previous report^31^, we observe Prox1-WT-mediated driving of cells to the SVZ in the absence of Ngn2. In addition, we have observed a positive effect in neurogenesis in the presence of Ngn2. Remarkably, both effects depend on sumoylation. Sall4a and Utf1, which were better expressed under proliferation conditions, also demonstrate to have a positive role in neurogenesis when sumoylated. Interestingly, we have detected *Sall4a* but not *Sall4b* transcript in P19 cells. While Sall4b has been more related to pluripotency, Sall4a has been related to differentiation and patterning, and more precisely to specific embryonic layers, in particular ectoderm^34^.

Sumo attachment to Utf1 seems to participate in at least two functional aspects: (a) modulating affinity for chromatin and (b) facilitating binding to Dcp1a. Thus, Utf1 adds to the list of transcription factors, sumoylation of which regulates its transcription activity. The effects of WT and KR proteins on the expression of bivalent genes were markedly different. The negative effect of KR Utf1 in neurogenesis correlates with the previously reported role of WT protein in stem cell differentiation and in transcriptional control of bivalent genes^42,44^. Sumo, by avoiding strong localization of Utf1 to regulated promoters, should facilitate partial access of repressors, but also would assure Dcp1a recruitment to promoters for decapping activity on leakage mRNAs. All this contributes to maintaining repression of bivalent genes, but in a poised state for rapid and effective activation upon induction of differentiation. Indeed, enhanced localization of the KR mutant to the chromatin might interfere with the localization of neurogenesis-related factors and contribute to the explanation of altered gene expression. Intriguingly, while a set of analyzed genes was negatively affected by the KR, the other set was negatively affected by the WT. Interestingly, this correlates with the previously reported effect of *Utf1* knockout on gene expression. Thus, those genes negatively affected by Utf1-WT (*Cdkn2a*, *Gata6*, *Hoxa1*, *Meis1*) are genes upregulated upon *Utf1* knockout, while most of those negatively affected by Utf1-KR (*Crabp2*, *Dll1*, *Neurod1*) are downregulated upon knockout^44^. Dcp1a interaction with Utf1 seems to be mediated by a SIM sequence conserved in vertebrate proteins. Since Utf1 evolutionarily appeared in eutherian mammals^43^, this raises the question of this SIM present in Dcp1a from non-eutherian mammals and other vertebrates. A possibility is that this SIM is used for interaction with other sumoylated proteins but also for interaction with an ancestor of eutherian Utf1 in other vertebrates.

Finally, Kctd15, preferentially expressed and sumoylated under differentiation conditions, appears to be involved in delaying neurogenesis. This suggests the involvement of Kctd15 sumoylation in controlling proper timing for neurogenesis progression, which otherwise might result in premature aberrant differentiation. Of note, we recently described another mechanism, also involved in delaying neurogenesis to avoid abrupt and abnormal differentiation^49^. In the embryo, non-sumoylated Kctd15 has been involved in inhibiting neural crest formation^30,38,50^, suggesting multiple developmental roles for Kctd15 sumoylation. Interestingly, Kctd15-KR showed neurogenic activity in the neural tube, since it promoted neurogenesis in the absence of Ngn2, pointing to a dominant-negative effect of the mutant protein, and indicating the probable involvement of progenitor-associated endogenous protein in preventing differentiation.

We cannot exclude additional modifications, other than sumoylation, targeting the same Lys residues studied in this work, but several lines of evidence strongly support the participation of sumoylation in neurogenesis. In-depth research in the near future will help to define the exact molecular mechanisms underlying the consequences of Sumo attachment to each specific factor and to groups of related factors.

## Supplementary information

Supplementary Figure and Table legends

Supplementary Figure S1

Supplementary Figure S2

Supplementary Figure S3

Supplementary Figure S4

Supplementary Figure S5

Supplementary Figure S6

Supplementary Table S1

Supplementary Table S2

Dataset 1
