# Supplementary material for: The Sumo proteome of proliferating and neuronal-differentiating cells reveals Utf1 among key Sumo targets involved in neurogenesis
Source: Cell Death Dis. 2021 Mar 22;12(4):305. doi: 10.1038/s41419-021-03590-2 (PMC7985304; doi:10.1038/s41419-021-03590-2)
Supplement: Supplementary file 1 — Supplementary Figure and Table legends [file 41419_2021_3590_MOESM1_ESM.docx]

**Supplementary Figure legends**

**Supplementary Figure S1. Gene Ontology analysis of genes coding for group II and group III proteins. a** Sector diagram indicating the number of proteins in groups I (red), II (green) and III (blue). **b** Gene Ontology analysis (GO) of genes coding for proteins in groups II and III (*p*-value<10^-2^). n, number of genes in each category.

**Supplementary Figure S2. Sall4 and Utf1 sumoylation in cells.** Sumoylation of expressed Flag-tagged Sall4a (**a**) and HA-tagged Utf1 (**b**) was tested in 293T cells through western blot with anti-Flag and anti-HA antibodies, respectively, in the absence (–) or the presence of expressed Ubc9 (+) and the indicated Sumo species (S1, Sumo1; S2, Sumo2). Absence of sumoylation of expressed sumoylation mutants was also tested (Flag-Sall4a 3KR and HA-Utf1 3KR). Black arrowheads indicate unmodified proteins while white arrowheads indicate sumoylated products. 20 µg of total protein were loaded per lane. **c** Position of the 5 Lys residues present in the mouse Utf1 sequence and mutated for the analysis in (B), are indicated.

**Supplementary Figure S3. Expression and cellular localization of WT and KR sumoylation mutants of Kctd15, Prox1, Sall4a, Trim24 and Utf1 in P19 cells. a** Mutated K (red) and surrounding sequences of selected proteins and references for previously described mutations. **b** Level of expression of expressed wild type (WT) or sumoylation mutant (KR) versions of Flag-tagged Kctd15, Sall4a and Trim24, or HA-tagged Prox1 and Utf1 proteins were determined in P19 cells by western blot. **c** Fold-overexpression was determined for each WT expression construct in western blot experiments of transfected (trf) and non-transfected (–) cells with specific antibodies against each protein. α-tubulin was determined as a loading marker. 20 µg of total protein were loaded per lane. **d** Cellular localization of Flag- or HA-tagged proteins analyzed by immunofluorescence with anti-Flag or anti-HA antibodies (red), respectively. Nuclei were visualized by DAPI staining (blue). Scale bar 25 µm. Transfection efficiency (flow cytometric determination of co-expressed GFP): 74.15 ± 2.8 (mean ± s.d., n=10).

**Supplementary Figure S4. Neurogenesis analysis in P19 cells.** P19 cells were transfected with expression constructs for NeuroD2 and the E12 co-factor together with expression constructs for WT or KR versions of the indicated proteins. Neurogenesis was evaluated 72 hours later by revealing the neuronal marker ßIII-tubulin (red). Transfected cells were visualized by expression of a GFP reporter. Nuclei were visualized by DAPI staining (blue). Scale bar 25 µm.

**Supplementary Figure S5. Neurogenesis analysis in embryos.** Neurogenesis in the developing neural tube was analyzed on embryos electroporated for 30 h with expression constructs for WT or KR versions of the indicated proteins, in the absence (empty) or the presence of a expression construct for the neurogenic factor Neurogenin2 (Ngn2). Mantle layer was visualized by revealing the marker ßIII-tubulin (red). Nuclei were marked by DAPI-staining (blue). Scale bar 50 µm.

**Supplementary Figure S6. Retinoic acid regulation of bivalent gene expression in P19 cells.** Levels of expression of the indicated bivalent genes were assessed in P19 cells by quantitative PCR under control proliferation conditions or after 48 hours of RA-treatment. Values are means ± s.d. from 3 independent experiments analyzed in triplicate. Statistical significance in relation to the control is indicated on top of each bar. Statistical significance was determined by the Student *t*-test. *p*<0.05*, *p*<0.01**, *p*<0.001***.

**Supplementary Table legends**

**Supplementary Table S1. Primers used for expression and ChIP analyses**

**Supplementary Table S2. Input and Sumo IP SILAC ratios of the 318 proteins with eligible SILAC IP ratios.** H/L, Heavy/Light SILAC ratios; exp, experiment; bold and shadow, proteins selected for validation; red, proteins without input ratios in both experiments (group I); green, proteins with an input ratio in at least one experiment and at least for one Sumo paralog, with IP/input relation > 1.5 or < 0.67 (group II); blue, rest of proteins (group III); NA, not a number.
