# Supplementary figures and images for: The Sumo proteome of proliferating and neuronal-differentiating cells reveals Utf1 among key Sumo targets involved in neurogenesis
Source: Cell Death Dis. 2021 Mar 22;12(4):305. doi: 10.1038/s41419-021-03590-2 (PMC7985304; doi:10.1038/s41419-021-03590-2)

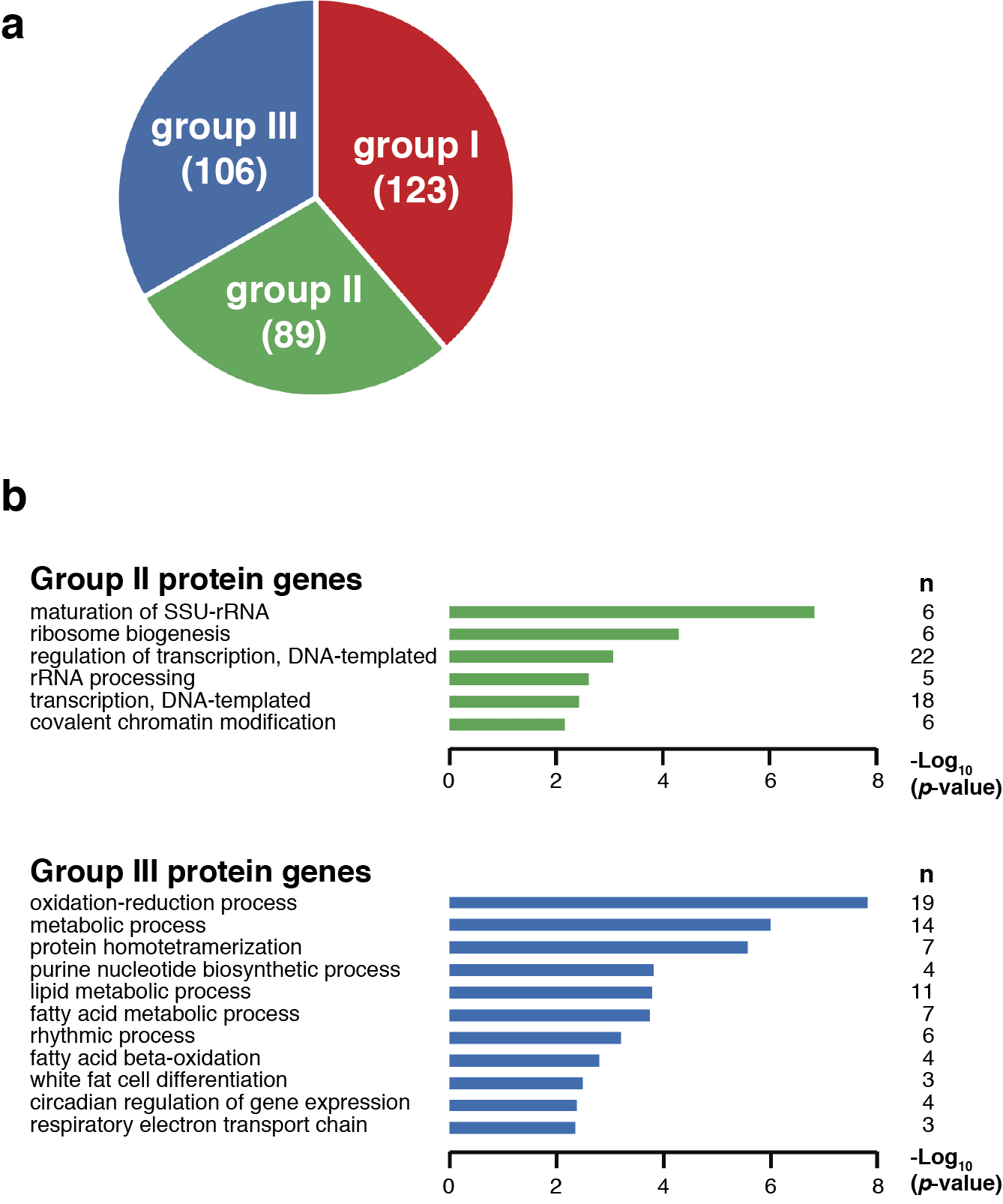

Supplement: Supplementary file 2 — Supplementary Figure S1 [file 41419_2021_3590_MOESM2_ESM.png]

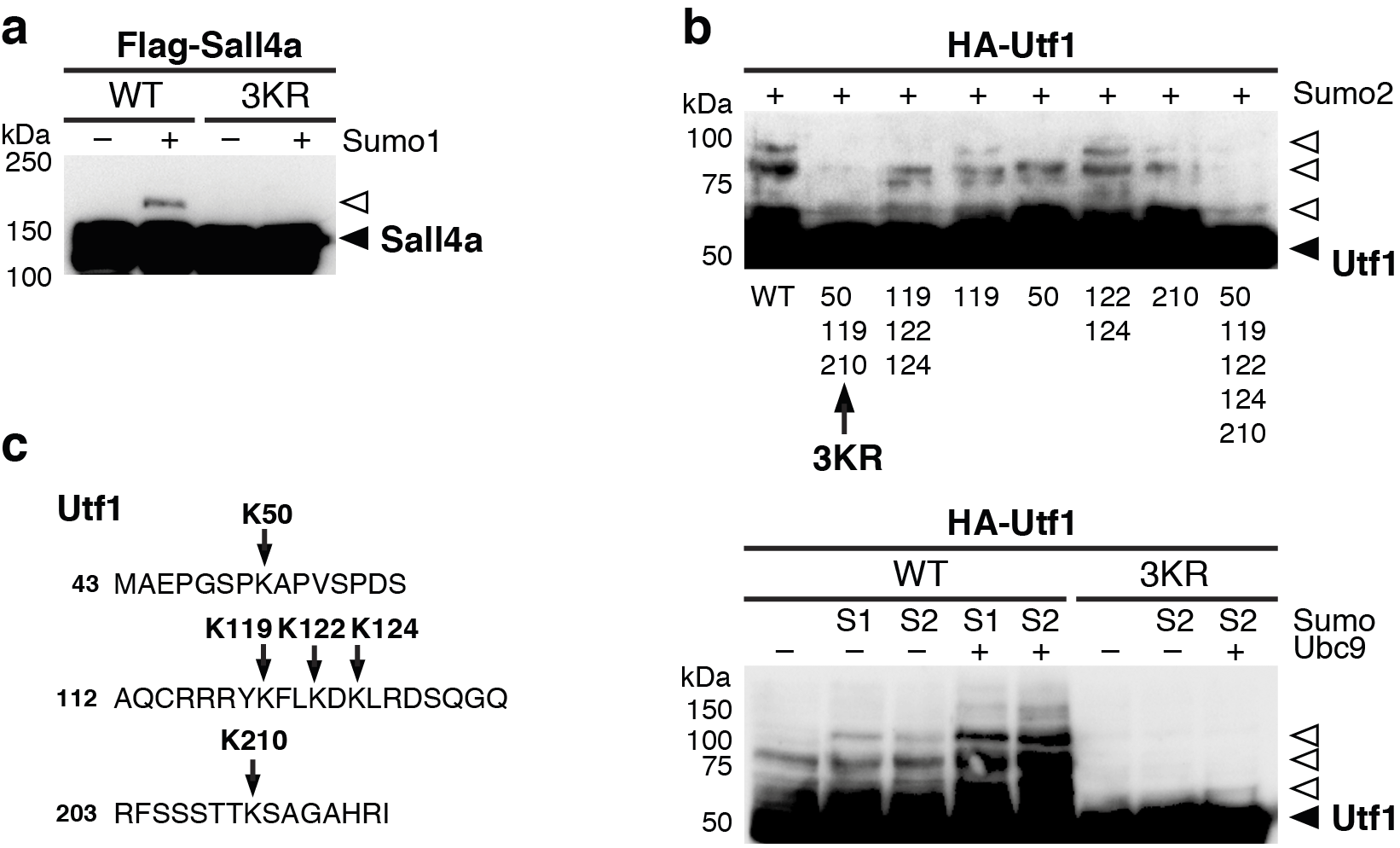

Supplement: Supplementary file 3 — Supplementary Figure S2 [file 41419_2021_3590_MOESM3_ESM.png]

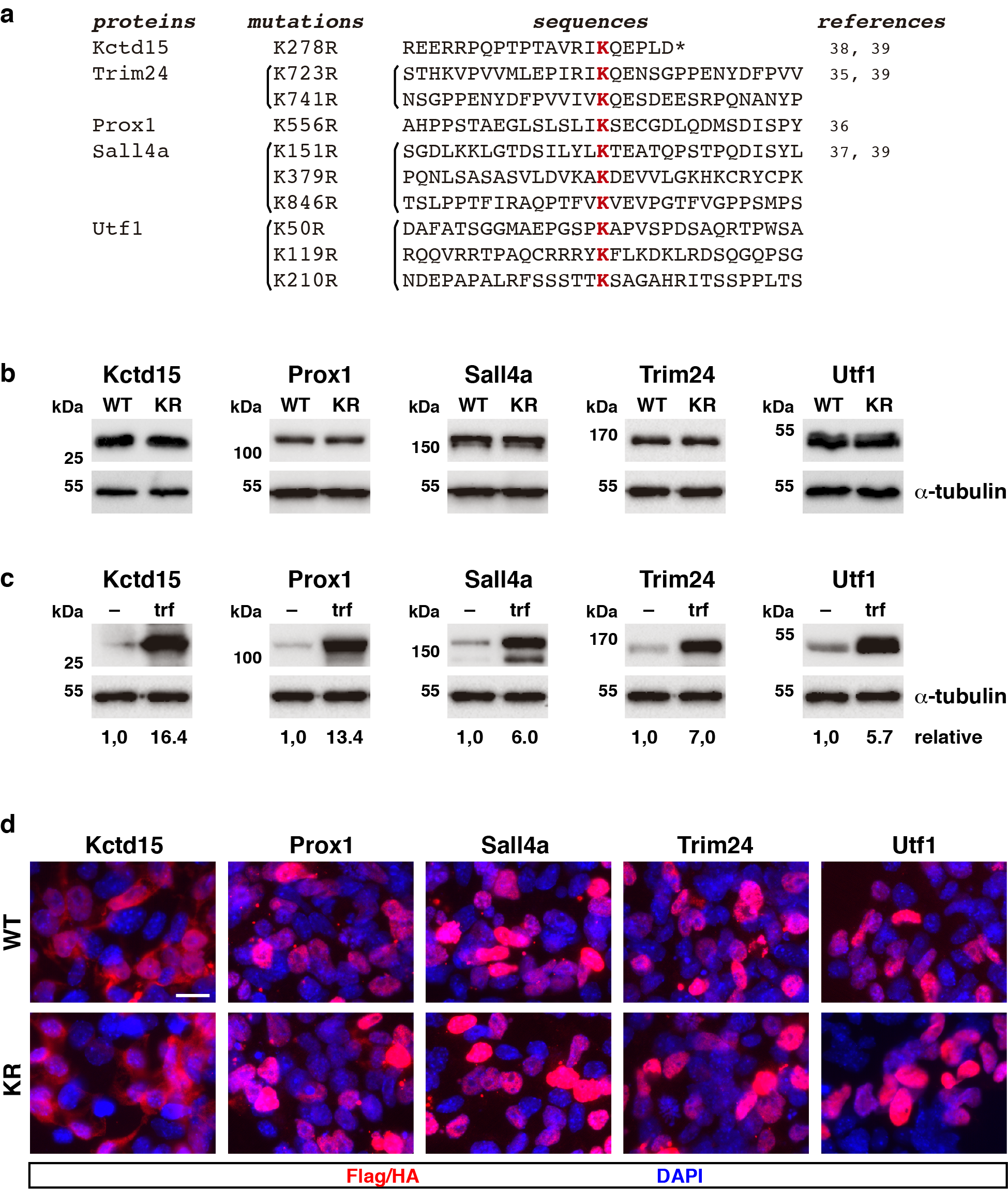

Supplement: Supplementary file 4 — Supplementary Figure S3 [file 41419_2021_3590_MOESM4_ESM.png]

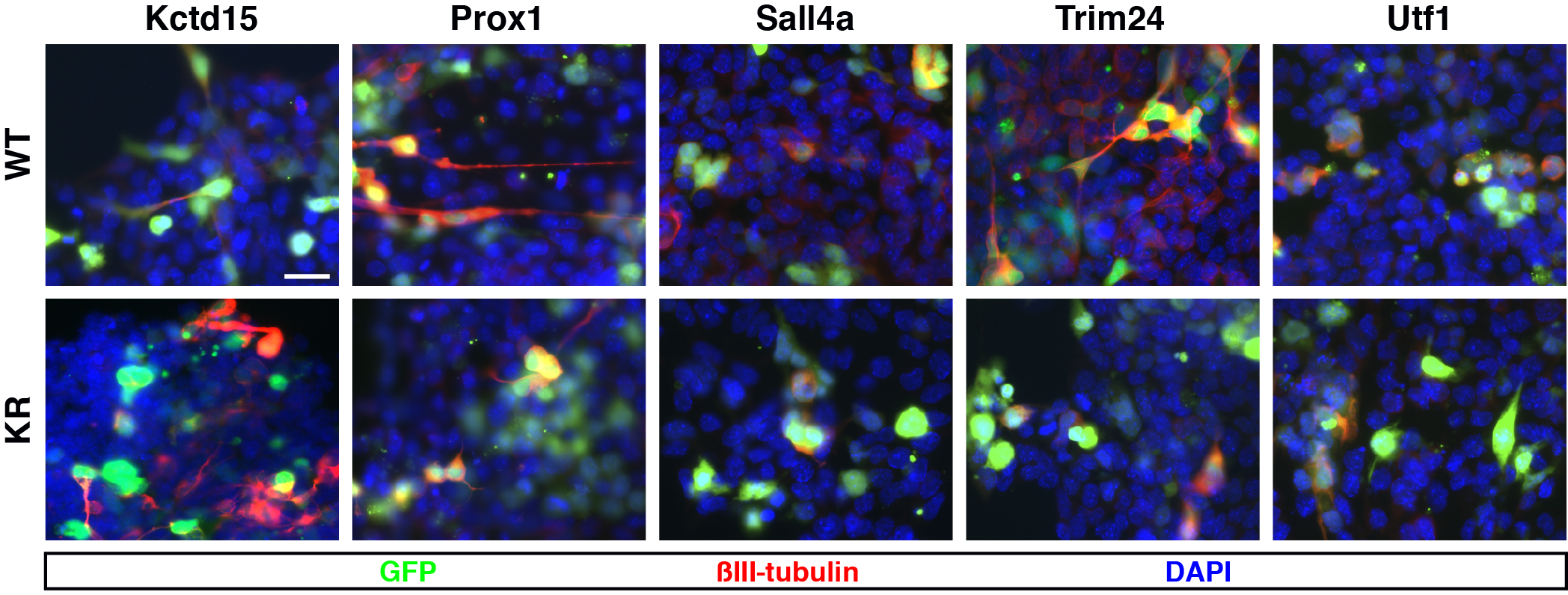

Supplement: Supplementary file 5 — Supplementary Figure S4 [file 41419_2021_3590_MOESM5_ESM.png]

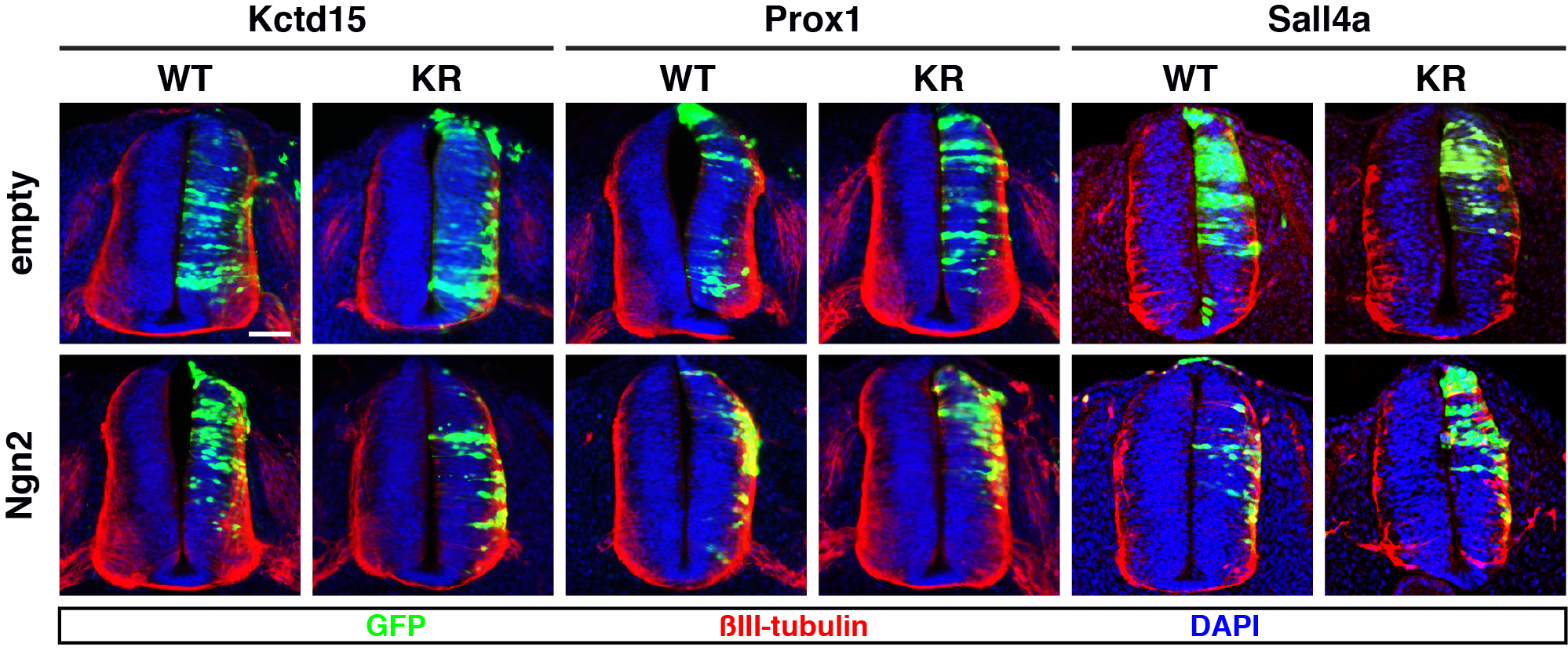

Supplement: Supplementary file 6 — Supplementary Figure S5 [file 41419_2021_3590_MOESM6_ESM.png]

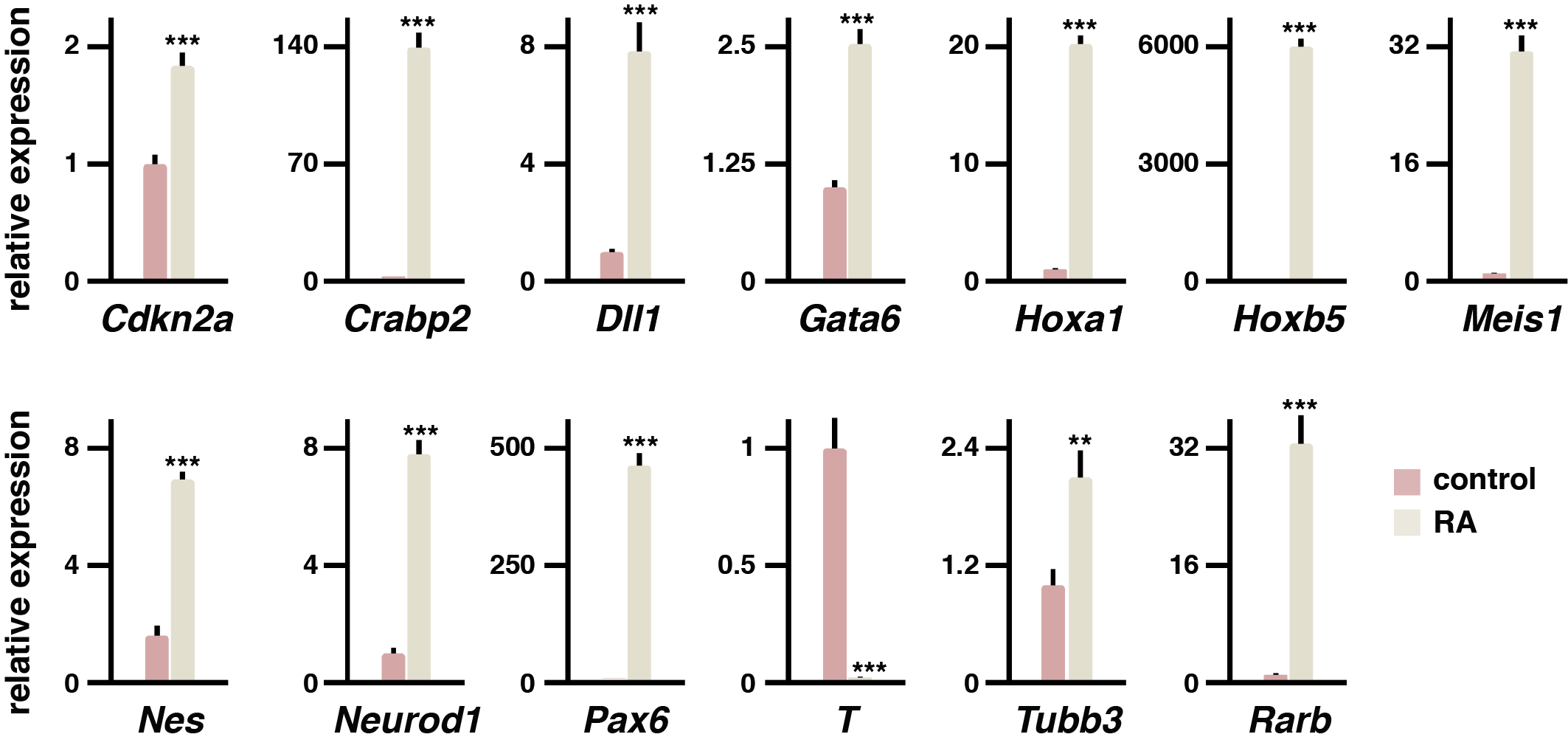

Supplement: Supplementary file 7 — Supplementary Figure S6 [file 41419_2021_3590_MOESM7_ESM.png]
