# Supplementary material for: The Sumo proteome of proliferating and neuronal-differentiating cells reveals Utf1 among key Sumo targets involved in neurogenesis
Source: Cell Death Dis. 2021 Mar 22;12(4):305. doi: 10.1038/s41419-021-03590-2 (PMC7985304; doi:10.1038/s41419-021-03590-2)
Supplement: Supplementary file 8 — Supplementary Table S1 [file 41419_2021_3590_MOESM8_ESM.docx]

**Supplementary Table S1. Primers used for expression and ChIP analyses**

| **expression** | **forward (5’ > 3’)** | **reverse (5’ > 3’)** |
| --- | --- | --- |
| *Ccne1* | GCAGCGAGCAGGAGACAGA | GCTGCTTCCACACCACTGTCTT |
| *Cdkn2a* | GCCGCACCGGAATCCT | TTGAGCAGAAGAGCTGCTACGT |
| *Crabp2* | CGATCGGAAAACTTTGAGGA | CACAGTGGTGGAGGTTTTGA |
| *Dll1* | TGGTCTCTGCCCTGCTGT | CCCCTTCTTGTTGACGAACT |
| *Gata6* | CAGCAGGACCCTTCGAAAC | CTCCGACAGGTCCTCCAAC |
| *Hoxa1* | GCAGACCTTTGACTGGATGAA | GAGCTGCTTGGTGGTGAAAT |
| *Hoxb5* | AGGGGCAGACTCCACAGATA | CCAGGGTCTGGTAGCGAGTA |
| *Meis1* | CTTTCCCAAAGTAGCCACCA | TGTGCCAACTGCTTTTTCTG |
| *Nes* | GATCGCTCAGATCCTGGAAG | TCAGGAAAGCCAAGAGAAGC |
| *Neurod1* | TCAGCATCAATGGCAACTTC | AAGATTGATCCGTGGCTTTG |
| *Pax6* | TACCAGTGTCTACCAGCCAAT | TGCACGAGTATGAGGAGGTCT |
| *Pou5f1* | CCAATCAGCTTGGGCTAGAG | CTGGGAAAGGTGTCCCTGTA |
| *Rarb* | CCTGCAGAAGTGCTTTGAAGTG | GCTTTCCGGATCTTCTCAGTGA |
| *T* | TCAAACTCACCAACAAGCTCA | CACGATGTGAATCCGAGGTT |
| *Tubb3* | ATGCCTCAGGAGGAAGTTGA | CTTGTTGGGAACCTGCATTT |
| *Rplp0* | CCAGGCTTTGGGCATCAC | CTCGCTGGCTCCCACCTT |
| **ChIP** |  |  |
| *Cdkn2a* | GACTCGGAGCAAGGGAAAC | TTTCGCTCCGGTTAACTTTC |
| *Hoxb5* | CTCTGAGCGGCTCTTACAGG | GCTGAGATCCATCCCATTGT |
| *Meis1* | CGTTCTCCAGCGGTCATAAATAG | AGGAAGGGAGGGAACAATGAG |
| *Neurod1* | GTCCGCGGAGTCTCTAACTG | GAACCACGTGACCTGCCTAT |
| *T* | CTTTGTTTCTTCCCGCTGAG | TAGGCAAACCTGGTCATTCC |
